# Transitivity, contextuality and decision making

**DOI:** 10.1098/rsta.2024.0373

**Published:** 2025-11-27

**Authors:** William H. Sulis

**Affiliations:** ^1^ CILab, Psychiatry and Behavioral Neurosciences, McMaster University, Hamilton, Ontario, Canada

**Keywords:** contextuality, transitivity, decision making, process algebra

## Abstract

In recent years there has been interest in the relationship between intransitivity and the presence of true (Type II) contextuality (contextuality with context independent marginals). The latter has been considered to be a *sine qua non* of quantum mechanics, although it has been observed in experiments on human decision making. Transitivity has long been viewed as essential to rational decision making although intransitivity appears to be ubiquitous in the natural world. An analogue of a *preference* graph is introduced for non-deterministic dynamical systems (of which decision making is an example) and used to identify several conditions which appear necessary for true contextuality to be present. Comparing the preference graph for the system against a reference preference graph formed from the marginals of the observables, one sees losses of options and intransitivity as necessary conditions.

This article is part of the theme issue ‘Quantum theory and topology in models of decision making (Part 1)’.

## 1. Introduction

Quantum systems and biological and social systems share many characteristics [1,2], which distinguish them from the inanimate systems studied within classical physics. The most important distinction, to my mind, is that quantum, biological and social systems share most of the characteristics of process [1–3], as described by Whitehead [4,5], as opposed to that of object, the focus for much of classical physics. Many authors have explored possible connections between these entities [6–11], whether in the physics or merely the formalism. Success of this undertaking depends upon an effective interpretation of quantum mechanics, which is singularly lacking, though there is no lack of contenders (Copenhagen, Bohmian, Transactional, Consistent Histories, Many Worlds). One group of interpretations that appears to offer much value for the biological and social sciences is that of the event-based interpretations [5].

An ‘event’ is a determinate fact of reality, which might be a measurement value, a behavioural act or an ‘actual occasion’ as proposed by Whitehead [4,5] in his theory of process. Events are circumscribed in time and space, possess determinate characteristics (meaning they can be agreed upon by multiple observers, recorded and referenced) and they constitute facts, meaning that they express logical truth.

Event-based interpretations view quantum mechanics as being essentially a theory that enables one to make predictions about the frequencies of occurrence of events, or of some characteristics associated with events. Neither the real nature of the system nor the surrounding world plays a role. It is fundamentally a set of tools (Schrödinger equation, Heisenberg matrices, Wigner’s phase space representation, Coecke’s diagrams, Chiribella’s information channels, Feynman path integrals) for the calculation of various probabilities concerning events and is not fundamentally a depiction of the systems which give rise to such events, nor of the mechanisms underlying the creation of events.

Event-based interpretations are explicitly epistemological in scope, eschewing ontology and avoiding the tendency to lapse into ‘quantum mysticism’, so prevalent with other interpretations. They appear to be more veridical—for example, the operator formalism is viewed as a mathematical tool for extracting information from an abstract mathematical wave function—neither the wave function nor the operators describe ‘reality’. Operators do not model actual measurement processes. That is an enormous undertaking (see [12,13]). Mathematical tools may be used whenever there is a homology between certain characteristics of the situation being modelled and the mathematics utilized in its description. Similar homology does not imply similar causality. One must always bear in mind the distinction between the representation of a situation and the situation being represented. Believing otherwise runs afoul of Whitehead’s Fallacy of Misplaced Concreteness.

## 2. The tossed coin metaphor

To better understand the relationships between systems, events and theory, I proposed a metaphor based on the tossing of a coin [2]. The tossed coin metaphor was introduced as a conceptual tool for stimulating thinking about certain ideas attributed to quantum mechanics, such as completeness and measurement, and its dogmatic attachment to Hilbert space formulations. It is not, however, meant to suggest a hidden variable type model for quantum mechanics as have other such models. Imagine a device for tossing coins and for measuring their outcomes, (H)eads or (T)ails. There is an impeller for propelling the coin vertically into the air, while a random generator imparts different chaotic spins to the coin. At the base of the device is a flat table with a momentum dissipating pad. This table is mounted on a vertical arm which allows it to rotate along a horizontal axis, thus changing the angle at which the table intersects the trajectory of the coin. After the coin is tossed, the angle can be rapidly adjusted at the will of the observer. When the coin lands on the table top, the coin quickly comes to a rest, either (H)eads up, or (T)ails up. The result is recorded, the coin returned to the impeller, and the experiment is run again.

The decision to measure and record *only* the final resting position of the coin is that of the observer. There is nothing special or fundamental about such a choice, but standard practice is that only the final resting position of the coin matters, either Heads up or Tails up. No other state constitutes a measurement. Thus, while the coin is in motion, no measurement takes place. In fact, no measurement value *can* be assigned because that requires halting its motion. Thus, there is either motion or a measurement in this experimental paradigm. Two measurements cannot be carried out immediately in sequence, because after the first measurement the coin is no longer in motion and so a second measurement yields a trivial result. This parallels the quantum mechanical situation of unitary evolution interspersed with non-unitary measurements. The measurement of H or T does not commute with the measurement of any other dynamical parameter for the same reason—if the measurement occurs first, the dynamics ceases. Note that the table cannot be set to two different angles simultaneously (cf. [14]).

Note that if a measurement is made with the table at an angle, the outcome will still always be either H or T, once the coin comes to a rest. Assuming a fair coin and a chaotic impeller, we can expect that H or T will appear with equal probabilities of 1/2. If these measurements are *all* that we are interested in, the Bernoulli theory gives us probability values of (1/2,1/2), providing a complete theory. It has been argued that no theory can improve upon the probabilistic predictions of quantum mechanics, therefore, quantum mechanics is effectively complete. But as this example shows, the notion of completeness is relative to whatever it is that we wish described. The Bernoulli theory is complete regarding the appearance of H or T, but not as regards the motion of the coin. Similarly, quantum mechanics may be complete as regards the appearance of measurement values, but it says nothing about a quantum system in its natural condition. *A priori*, there is no reason to believe that the choice of ‘mangle of practice’ [15] associated with the standard physical observables constitutes the totality by means of which we can interrogate Nature. As Hamlet said, ‘There are more things in Heaven and Earth, Horatio, than are dreamt of in your philosophy’ (Act I, Scene 5, Hamlet, Wm Shakespeare).

Several things can be noted.

(i) The tossed coin is a *process—*it is a generator of conditions for generating a measurement value of H or T, *if* a measurement is carried out. H and T are not properties of the tossed coin, but potentiae determined by the tossed coin if it interacts with a measurement apparatus. Bohr was correct in stating that a measurement must always be referenced to the system and the measurement apparatus, but that does not imply that the act of measurement somehow *creates* the tossed coin, it merely co-creates the measurement value, H or T. But these are not properties of the tossed coin, merely potentia associated with the coin.
(ii) There are no ‘hidden variables’ in the sense that from a specific value of an instantaneous state of the tossed coin, one cannot determine a value of a measurement or a probability of a measurement (here the probabilities are actually independent of any instantaneous state but that is not true of systems generally). Nevertheless, there are clearly hidden variables that participate in the ‘dynamics’; of the tossed coin, and this dynamics together with the ‘structure’ of the situation, serve to define a probability of measurements, but this is not connected to the *value* of any specific hidden variable. The Bell definition of a hidden variable does not pertain here.
(iii) The probability is meaningless unless something actually happens.
(iv) The description of the sequences of measurement events by means of the (1/2,1/2) probability distribution provides a complete description of the dynamics of these events, *provided that the only events that one is interested in are these sequences of H andT* .
(v) If one is interested in any other aspect of the tossed coin scenario, then one must appeal to an examination of the actual dynamics of the coin as it is tossed. For that, one requires a different set of tools, different apparatus, different paradigms, and so on. Different strategies for interrogating the situation are required.
(vi) Ascribing a notion of a ‘state’ to the coin and labelling such a state H or T is entirely misleading. Such a determination is meaningful *only* in the context of a measurement situation, and in such a situation the coin is undergoing no dynamics at all—it is at rest. These measurements are not conditions of the coin itself—they are artefacts of our method of measurement. In flight, the coin is never in a state of H or T, even instantaneously. Measurements do not exist while the coin is in flight, but the coin itself exists.
(vii) The tossed coin violates Leggett and Garg’s definition of macrorealism [16] because it is never in a state of H or T during its motion (so it does not possess one of its attributes at each point of time, hence not macrorealism) and it cannot be measured without disturbance (hence no non-invasive measurability). Nevertheless, I doubt that anyone would argue that a tossed coin is not macroscopically real.
(viii) The above shows that the tossed coin does not express counterfactual definiteness. Between measurements it does not take on definite values of possible measurements. It possesses no measurement values *until* a measurement is made. Yet it is manifestly real, nonetheless.
(ix) No mystical ‘collapse’ of reality takes place when a measurement occurs. Even if one might consider the probability distribution to serve as a state representation for the tossed coin, there is no collapse of this state. All that happens is a perfectly understandable interaction between a measurement apparatus and the tossed coin, which results in that eventual state which corresponds to a measurement. An outcome occurs. Period. The probability of the outcome is given by the distribution function. At rest the distribution function is now different (concentrated on the single outcome) because the coin is at rest. The coin still exists. The probability distribution function supervenes on the dynamics of the system, not the other way around.
(x) The peculiarity of spin measurements is resolved. Note that the measurement platform can be set at any angle, but one will only ever record an H or a T, never a fractional value. This again is an artefact of the measurement procedure, not a property of the tossed coin.

An event interpretation leaves the door open to utilize the quantum formalism to determine the probabilities of events in other settings without having to justify or explain away the baggage of quantum physics.

## 3. Transitivity

Mathematical constructs have been widely used in decision theory, especially in notions of preference, transitivity utility. Transitivity (meaning in a relation R, if xRy and yRz then xRz) is a very useful property from a mathematical perspective. As Tversky [17] points out, a major reason for requiring preferences to obey transitivity is that it is a necessary condition for the existence of an ordinal (utility) scale, and a sufficient condition provided that the number of alternatives is countable. The existence of such a scale is useful for the development of mathematical models of decisions involving preference, but it is far from clear that actual people or animals rely on or manifest such scales in their actual decisions. The introduction of concepts such as utility, or the imposition of formal constraints such as rationality, of which transitivity forms one condition, appears to be driven by the desire to utilize certain kinds of mathematics, and not based on empirical observations. Wigner [18] famously wrote a paper titled ‘The Unreasonable Effectiveness of Mathematics in the Physical Sciences’, but I was taken by a paper of Velupillai [19] titled ‘The Unreasonable *In*effectiveness of Mathematics in Economics’. Transitivity in decision making provides a case in point. Early writings on the role of transitivity emphasized logical, rational or mathematical ideas, with empirical studies appearing much later [20].

If only a single attribute is used to make a preference choice, then it does indeed seem reasonable, if not necessary, to assert that such choices should exhibit transitivity. Tversky [17], however, pointed out that many, if not most, real-world preferences are based upon multiple attributes, and suggested modelling preferences with a semiorder [21], rather than a linear order.

Formally, one assumes the existence of a set of n attributes for each alternative, and for each attribute $x_{i}$, a function (perhaps a utility function) $u_{i}$ which assigns a value $u_{,}(x_{i})$ to each attribute. The value can be ordinal or numerical. As an example, given two alternatives $x=\left(x_{1},\ldots ,x_{n}\right)$ and $y=\left(y_{1},\ldots ,y_{n}\right)$, a preference may be determined additively, meaning x<y iff $u(x)=\sum_{i}u_{i}(x_{i})<\sum_{i}u_{i}(y_{i})=u(y)$. These models are convenient mathematically, but it is not clear that these scales, or functions $u_{i}$ or $\phi_{i}$ actually exist. Moreover, a presumption is that these values are fixed attributes of these alternatives. If absolute scales exist, then as Butler & Progrebna [22] argue, ‘Transitivity must hold either if a value attaches to each option without reference to other alternatives (choice-set independence), or if an equivalent value results after comparing and contrasting the attributes of the available choice options’.

The appearance of transitivity appears to be strongly tied to additive strategies. Other strategies may manifest transitivity only under restricted conditions, which suggests that transitivity should not be considered as universal, or essential. Anand [23] has argued that intransitivity need not necessarily be irrational. In social insect colonies, individual workers might display evidence of non-rational decision making or intransitive preferences, but the social insect colony as a whole may compensate and effect transitive preferences and rational decision making [24,25]. Context plays a significant role in determining whether decision making follows the constraints of rationality, or veers into the non-rational [26–29]. In [30], I presented a simple set of ice cream preferences based on differences in pleasure–pain propensities together with a threshold criterion which is intransitive, not lexicographic and not additive, yet still principled.

There have been many studies demonstrating the occurrence of intransitivity in decision making [31,32]. Nevertheless, several authors continue to deny the existence of intransitive preferences [33–36]. Some, such as Regenwetter, claim that transitivity is in fact a universal phenomenon and any appearance of intransitivity is a sign of methodological error [33,34]. Adherence to transitivity has some of the features of a cognitive bias.

Transitivity is not universal in the natural world. Far from it. Consider three very simple relations. The first is nearness. A is said to be near to B if their distance is less than 1 mile. Clearly then, if A,B,C lie on a straight line and A is near to B, and B is near to C, A need not be near to C, by simply taking the distance ρ(A,B)=ρ(B,C)=1*,* while ρ(A,C)=2. Another example is ‘next’, as in next step, or next action. Suppose one has a door with a handle lock and a hook lock. Suppose one starts with the handle and hook locks set. Then ‘unlock latch’ is next to ‘unlock handle’ and ‘open door’ is then next to ‘unlock latch’, but ‘open door’ is not next to ‘unlock handle’, unless ‘unlock latch’ has already transpired. From the starting position, ‘open door’ cannot be next to ‘unlock handle’. A third example is ‘direct flight to’, where clearly one can have a direct flight from A to B and from B to C, but there need be no direct flight from A to C. In mathematics it is well known that one can take a relation or a graph and form the transitive closure, so that if one has aRb and bRc, then one adds aRc. Denote the transitive closure of R by $R^{tc}$. But $R^{tc}$ is a different relation from R, not just in terms of set content but in terms of meaning as well. For example, the transitive closure of the relation ‘direct flight to’ is simply the relation ‘flight to’.

A major failing of decision theory, to my mind, is that it places decision making within an abstract, purely cognitive setting, ignoring decades of research in embodied cognition. I prefer french fries to pasta, but after once spending a week at the end of season at a resort when every meal came with french fries, I found myself desperate for anything else, including pasta. Real preferences are contextual, expressed by a living entity with a living body, which has its own say. An interesting experiment [32] examined neural sources of intransivity. Moreover, outcomes of experiments on decision making appear to depend upon the experimental paradigm being utilized, thus demonstrating contextuality at the level of experimental design [37].

## 4. Contextuality

In recent years there has been interest in the relationship between decision making and contextuality. There has also emerged some interest in the relationship between intransitivity and contextuality [38,39]. There are highly abstract approaches to contextuality based upon operators in Hilbert space [40], POVMs [41], sheaf theory [42], category theory [43] but I am partial to the approach of Dzhafarov since it connects more directly to actual experiments. There are two principal forms of contextuality. The most ubiquitous form, termed inconsistent connectedness in the Contextuality by Default approach of Dzhafarov [44,45] or Type I Contextuality [1], is prevalent in the life sciences [46], decision theory [37] and in the construction of science [15].

A second form of contextuality, termed True Contextuality by Dzhafarov [44] or Type II Contextuality [1], is more stringent, and said to distinguish quantum from classical mechanics, though it has been observed in classical settings involving decision making [6,47–51]. This is contextuality determined by violations by correlations of various inequalities (Bell [52], Leggatt-Garg [16], CHSH [53], Gisin [54], Mann [55]).

Dzhafarov has developed an analogue of the CHSH inequality [44] in his Contextuality by Default approach [44]. Each random variable is identified by the property q that it measures and the context a within which it is measured, and so each random variable is denoted as $R_{q}^{a}$ and a collection of random variables can be organized in the form of a matrix. For example, the CHSH situation can be viewed as a cyclic 4 system, that is, as a system of four random variables which can be arranged in an array having the following form:


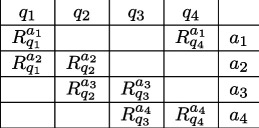


Each row in the matrix is termed a *bunch*, and each column a *connection*. Since the random variables in a bunch have the same context, they are presumed to possess a joint distribution. The random variables in a connection have different contexts and so cannot be assumed, *a priori*, to possess a joint distribution. If they do, they are called consistently connected. If they do not, they are called inconsistently connected.

Dzhafarov [44] argued for a general inequality, given here for cyclic systems of order n. This inequality is ΔC=


$$\begin{array}{r} s(\pm <R_{q_{1}}^{a_{1}}R_{q_{n}}^{a_{1}}>\pm <R_{q_{1}}^{a_{2}}R_{q_{2}}^{a_{2}}>\pm <R_{q_{2}}^{a_{3}}R_{q_{3}}^{a_{3}}>\cdots \pm <R_{q_{n-1}}^{a_{n}}R_{q_{n}}^{a_{n}}>)-\\ (n-2)-\sum_{1}^{n}|<R_{q_{i}}^{a_{i}}>-<R_{q_{i}}^{a_{i+1}}>|\leq 0, \end{array}$$

where s means the maximum taken over all combinations of terms such that the number of minus terms is always odd, and


$$<R_{q_{i}}^{a_{n}},R_{q_{j}}^{a_{n}}>=\int R_{q_{i}}^{a_{n}}R_{q_{j}}^{a_{n}}d\mu =\int E(i,j;n)d\mu_{a_{n}},$$

where


$$E(i,j;n)=R_{q_{i}}^{a_{n}}R_{q_{j}}^{a_{n}}$$

and $\mu_{a_{n}}$ is the probability measure for the joint probability distribution for the random variables in the context $a_{n}$. The first term is the CHSH term, the second compensates for the degree of cyclicity, while the third term compensates for inconsistent connectedness. The system exhibits Type II contextuality if the inequality is violated for some set of observables. The case of 4-cycles exactly matches that of CHSH.

A simple argument [2,14] shows that if this inequality is violated, then a single joint distribution covering all of the relevant variables $q_{1},\ldots q_{n}$ does not exist. I will consider the case of 4-cycles as the extension to the general case which follows in a straightforward (if lengthy) manner.

Shimony [56] shows that the function of four variables $a,a^{\prime },b,b^{\prime }$


$$chsh(a,a,b,b^{\prime })=ab+a^{\prime }b+ab^{\prime }-a^{\prime }b^{\prime },$$

where the range of values for each variable [−1,1] has values in the range [−2,2]. Therefore if there exists a probability measure μ on the simplex $[-1,1{]}^{4}$, then


$$|\int chsh(a,a^{\prime },b,b^{\prime })d\mu |\leq \int |chsh(a,a^{\prime },b,b^{\prime })|d\mu \leq 2\int d\mu \leq 2.$$

Now consider the function ichsh=a+b+c−d on $[-1,1{]}^{4}$ where the variables a,b,c,d are independent of one another. Then this function clearly takes values in the range [−4,4]. The function chsh cannot take these extremal values {−4,4} because each term in *chsh* is a product of two variables, effectively correlating or entangling them [14].

If there is a joint probability measure μ for *all* of the random variables in the formula, which effectively means that context may be ignored, then replicating the analysis for the *chsh* formula, it follows that

| [E(1,2;2) +E(2,3;3) +E(3,4;4) −E(1,4;1)]dµ|≤

|[E(1,2;2) + E(2,3;3) + E(3,4;4) − E(1,4;1)]|dµ≤2 dµ=2.

In the event that a joint probability measure such as μ does not exist, then it is incorrect to integrate over the function E(1,2;2)+E(2,3;3)+E(3,4;4)−E(1,4;1) on the whole. Instead, we must integrate each term separately, using the joint probability distribution appropriate for each pair of random variables. In this case we obtain


$$\int E(1,2;2)d\mu_{1,2;2}+\int E(2,3;3)d\mu_{2,3;3}+\int E(3,4;4)d\mu_{3,4;4}-\int E(1,4;1)d\mu_{1,4;1}.$$

This is the same as for the function *ichsh* since each integral extremizes to {−1,1}, so that

| | E(1,2;2)dµ1,2;2 + E(2,3;3)dµ2,3;3 + E(3,4;4)dµ3,4;4 − E(1,4;1)dµ1,4;1|≤

E(1,2;2)dµ1,2;2| + | E(2,3;3)dµ2,3;3| + | E(3,4;4)dµ3,4;4| + | E(1,4;1)dµ1,4;1|≤4.

Thus, if the *chsh* inequality is violated, it must be the case that there can be no single joint distribution for *all* of the random variables. This applies by extension to Dzhafarov’s inequality. This also implies that the appropriate measure for each term cannot be a simple product of the marginal probabilities for each of the involved random variables, because in that case one could form a joint probability by taking the product of all of the marginals. Hence, if the inequality is violated, one of these probability measures cannot be a simple product of marginals. I will need this observation in the next section.

## 5. Process

For several years I have been exploring the foundations of, and attempting to formulate, a realist version of, quantum mechanics through the lens of Whitehead’s Process Theory [4] and the Process Algebra [57]. The Process Algebra is an attempt to formalize some of the ideas of process developed by Whitehead [4]. Details can be found in [2,57]. Heather & Rossiter [58] also attempted to formulate Whitehead’s theory, within the domain of category theory, but their theory is high level, whereas the Process Algebra was explicitly developed to enable calculations to be carried out. Process is conceived as a *generator* of primitive information laden events, informons (akin to Whitehead’s actual occasions), which are created in a generation, persist only long enough to propagate their information to the next generation, and then fade away to make way for the next generation. Informons may also be thought of as events within an event interpretation. A process creates only a finite number of informons in a generation. Thus, they create discrete spaces. Continuous spaces and entities can be formed through the procedure of interpolation, of which there are many forms depending upon the topological and metric properties of the space of informons [57,59–61]. Processes interact to form nexus, which can be represented by labelled graphs. Interactions are classified on the basis of three characteristics:

(i) The relative timing of the generation of informons: *Sequential* or *Simultaneous*
(ii) The flow of information from prior to nascent informons: *Exclusive* or *Free*,
(iii) The relationship between processes: *Independent*, *Coupled*, *Entangled* or *Interacting*.

There are 11 distinct operations in the Process Algebra, but here we need only focus on the exclusive, interactive product ⊠, which represents interactions between two distinct entities whose informons do not share information, as in a measurement situation.

A process will generally produce a large, but finite, number of informons within a single generation. A history of these generations forms a causal tapestry, which can be represented as a finite graph [2]. Processes are non-deterministic: allowing the process to act again starting from the same initial conditions will generally result in a different set of informons in the generation and so a different causal tapestry (graph). Only the relative relationships between these informons is important, and so the space of graphs will be large, but again finite, and one can, in principle, determine relative frequencies of informons, and thus a probability structure. We may think of a process, given an initial condition, as making decisions regarding which state or states should ensue. We may interpret the associated probabilities as giving the *preference* that the process has for a particular state given some initial condition. While all measurements are events, triggered by informons, not all informons are measurements.

We can create a *preference graph* as follows: If Π is a process and I some initial condition, then the preference graph p(Π,I) is simply defined as having as vertices the set of all possible informons, N, and edges are defined by x→y iff f(y)>f(x), where f is the relative frequency of occurrence of the event in the process covering graph of Π with initial condition I. It is a directed graph.

The situation that is of interest involves a system, here generated by some process Π, and two observers, A and B, represented by processes $O_{A}$ and $O_{B}$, respectively. We are interested in correlations between observations of these two observers on the system. This is the situation of entanglement in quantum mechanics, where the system is viewed as comprising two separate particles, usually photons or electrons, with some ‘spooky action at a distance’ acting between them. The two particles are viewed as somehow separate, with each observer acting solely upon one of the particles. This is made more justifiable by the fact that the probability distribution of measurements as observed by each observer is the same as in the case in which the two particles are entirely independent. It is this that creates the greatest perplexity and part of the reason why the assumption of consistent connectedness is important in asserting the presence of Type II contextuality. In the Process Algebra this is represented by $O_{A}⊠\Pi ⊠O_{B}$. In the process perspective, however, the system process Π is to be treated as a complete whole.

If we describe these two situations within the Process Algebra, letting $\Pi_{1}(a),\Pi_{2}(b)$ be the processes for the two particles (a,b denote states), then in the free case we would write


$$O_{A}⊠\Pi_{1},\Pi_{2}⊠O_{B},$$

while in the entangled case we have


$$O_{A}⊠[(\Pi_{1}(a)⊠\Pi_{2}(b))⊕(\Pi_{1}(b)⊠\Pi_{2}(a))]⊠O_{B}=$$

$$[O_{A}⊠(\Pi_{1}(a)⊠\Pi_{2}(b))⊠O_{B}]⊕[O_{A}⊠(\Pi_{1}(b)⊠\Pi_{2}(a))⊠O_{B}].$$

In general, it is not true that


$$O_{A}⊠(\Pi_{1}⊠\Pi_{2})⊠O_{B}=(O_{A}⊠\Pi_{1})⊠(\Pi_{2}⊠O_{B})=(O_{A}⊠\Pi_{1})⊗(\Pi_{2}⊠O_{B}).$$

*A priori*, there is no reason to believe that the entangled case should reduce to the free case, or that the same probability distribution can be used to describe the joint random variables. In the application of classical Kolmogorov probability theory, it is usually assumed that a joint probability distribution can be found which will encompass both cases, especially as the marginal probabilities of the two random variables are identical. That this is not the case in general was shown by Vorob’ev [62] decades ago. Suppose that one is given three two-dimensional random variables $(X_{1},Y_{1}),(X_{2},Z_{2}),\left(Y_{3},Z_{3}\right)$ and the distribution functions of $X_{1}$ and $X_{2}$, $Y_{1}$ and $Y_{3}$, $Z_{2}$ and $Z_{3}$ coincide, respectively. Is there a three-dimensional random variable (X,Y,Z) whose two-dimensional projections have distributions matching those of $\left(X_{1},Y_{1}\right)$,$\left(X_{2},Z_{2}\right)$,$\left(Y_{3},Z_{3}\right)$? The answer, in general, is no. Examples of collections which do not in general possess joint measures include the complex $G_{n}$ of all proper subsets of a set of n elements for n≥3, and the complex $Z_{n}$ consisting of skeletons of the form $\{a_{1},a_{2}\},\{a_{2},a_{3}\},\ldots ,\{a_{n-1},a_{n}\},\left\{a_{n},a_{1}\right\}$ for n≥3. In particular, note that taking a triple of probability measures and then considering correlations involving pairs of the individual event spaces of the form $(a_{1},a_{2}),(a_{2},a_{3}),\left(a_{3},a_{1}\right)$ runs afoul of his result.

## 6. Process, contextuality and transitivity

Let us apply these ideas to the situation described by $O_{A}[a]⊠\Pi ⊠O_{B}[b]$ for some observed process Π, observers $O_{A},O_{B}$ and observables a,b, respectively. I will focus on the situation in which the marginal probabilities are consistently connected, which is the setting for Type II contextuality. Since the marginals are consistently connected, we do not need to reference their contexts and so we may simply denote their probability measures as $p_{a},p_{b}$ for observables a,b, respectively. The probability for the context of joint measurement of a,b is denoted by $p_{a,b}$*,* while that formed by the product of the marginals is denoted by $p_{ab}$ where $p_{ab}(x,y)=p_{a}(x)p_{b}(y)$.

Let I be *a* set of *alternatives* and p a *probability* distribution on I. The *null set* of p, $N_{p}=\left\{x\in I|p(x)=0\right\}$. The *weak preference graph* of p has as vertex set $I/N_{p}=\left\{x\in I|p(x)\neq 0\right\}$ *and* an edge x→y iff p(x)≤p(y). If p(x)=p(y), then there will be two edges x→y and y→x. The subgraph consisting of all such pairs and their edges is called the *indifference (sub)graph* of p. The *strong preference (sub)graph* of p consists of all pairs x,y such that p(x)<p(y) together with their edges.

The weak preference*,* indifference and strong preference graphs are totally connected graphs because we can always compare the probabilities of the vertices and place either one or two edges linking them. They are all transitive graphs as well, since the ordering is based on the usual ordering of the reals.

The first thing to note is that the null set of $p_{a,b}$ contains the null set of $p_{ab}$. For suppose that (x,y) lies in the null set of $p_{ab}$. Then $p_{a}p_{b}(x,y)=p_{a}[x]p_{b}[y]=0$*,* so that either $p_{a}[x]=0$ or $p_{b}[y]=0$. Assume the former. Since the marginals of $p_{a,b}$ are consistently connected and match those of $p_{ab}$, it follows that $\sum_{y}p_{a,b}(x,y)=p_{a}[x]=0$*,* which implies that $p_{a,b}(x,y)=0$ for all y, so that in particular, $p_{a,b}(x,y)=0$ and (x,y) lies in the null set of $p_{a,b}$.

If $p_{a,b}\neq p_{a}p_{b}$, then what does this imply for their preference graphs? The issue for contextuality is whether or not the departure from the product substantially changes the expectation value of the observables. The inequality ΔC can always be constructed in which the term in question serves as one of the negative terms in the formula. Minimizing this term or reversing its sign will reduce its effect upon the positive terms. This will increase the value of the formula and if it can be increased enough, its value could exceed the bound and the system would manifest true contextuality.

There are two main possibilities: either the two preferences are isomorphic, or they are non-isomorphic. Suppose, first, that the two preference graphs are not isomorphic. They can be different in one of several ways

(i) The null set of $p_{a,b}$ is strictly larger than that of $p_{a}p_{b}$. This can occur in circumstances in which the measurement of certain pairs of observables is forbidden or impossible—for example owing to non-commutativity of observables in quantum mechanics, or to an obstruction in the setting (real entities have physical extension). This results in a loss of completeness. Such is the case in entanglement, where the process of entanglement precludes the possibility of certain combinations of observables (for example, state pairs (0,1),(1,0) are permitted while (1,1),(0,0) are not).
(ii) The indifference set is smaller. This can only occur if the probabilities between two events $\left(x^{\prime },y^{\prime }\right)$ and $\left(x^{\prime \prime },y^{\prime \prime }\right)$ are different in $p_{a,b}$ but equal in $p_{a}p_{b}$. In that case there is a loss of two edges, $(x^{\prime },y^{\prime })\to \left(x^{\prime \prime },y^{\prime \prime }\right)$ and $(x^{\prime \prime },y^{\prime \prime })\to \left(x^{\prime },y^{\prime }\right)$*,* from the indifference graph of $p_{a}p_{b}$, and the addition of one edge, either $(x^{\prime },y^{\prime })\to \left(x^{\prime \prime },y^{\prime \prime }\right)$ or $(x^{\prime \prime },y^{\prime \prime })\to \left(x^{\prime },y^{\prime }\right)$ to its strong preference graph. Note that although the preference graphs for $p_{a,b}$ and $p_{a}p_{b}$ *are* transitive themselves, these changes can result in intransitivity from the viewpoint of the graph for $p_{a}p_{b}$. That is, if we make the corresponding change in the graph for $p_{a}p_{b}$ without the compensating changes seen in $p_{a,b}$*,* we may observe a breach of transitivity.
(iii) The indifference set is larger. This can only occur if the probabilities between two events $\left(x^{\prime },y^{\prime }\right)$ and $\left(x^{\prime \prime },y^{\prime \prime }\right)$ are different in $p_{a}p_{b}$ but equal in $p_{a,b}$*,* or where two elements that have different probabilities in $p_{a}p_{b}$ have the same probability in $p_{a,b}$. In that case an additional arrow in the reverse direction would be added, which would violate transitivity in $p_{a,b}$. In the former case, there is a gain of two edges, $(x^{\prime },y^{\prime })\to \left(x^{\prime \prime },y^{\prime \prime }\right)$ and $(x^{\prime \prime },y^{\prime \prime })\to \left(x^{\prime },y^{\prime }\right)$*,* for the indifference graph of $p_{a}p_{b}$, and the loss of one edge, either $(x^{\prime },y^{\prime })\to \left(x^{\prime \prime },y^{\prime \prime }\right)$ or $(x^{\prime \prime },y^{\prime \prime })\to \left(x^{\prime },y^{\prime }\right)$ from its strong preference graph. Again, if we make the corresponding change in the graph for $p_{a}p_{b}$ without the compensating changes seen in $p_{a,b}$*,* we may observe a breach of transitivity.
(iv) The null and indifference graphs are identical, but the strong preference graphs differ. This can only occur if an edge in $p_{ab}$, say x→y is reversed in $p_{a,b}$*,* so y→x. This can obviously result in intransitivity.

Assume now that the preference graphs are isomorphic. Then the null sets are identical. Since they do not contribute to the calculation of expectation values, we shall focus our attention on the indifference and strong preference subgraphs, which must be isomorphic. Since the graphs are isomorphic, so are the order relations, hence for all $(x,y),\left(x^{\prime },y^{\prime }\right)$, $p_{a,b}(x,y)\leq p_{a,b}(x^{\prime }y^{\prime })$ iff $p_{ab}(x,y)\leq p_{ab}(x^{\prime },y^{\prime })$. This is also true for the marginals. The vertices in the indifference graph can be partitioned into equivalence classes in which each vertex is assigned the same probability. These probabilities may change under an isomorphism, but the partitioning is invariant. We may choose one representative (x,y) from each equivalence class and allow it to stand for the remainder and assign it the probability $np_{a,b}(x,y)$ or $np_{ab}(x,y)$ where n is the size of its equivalence class. Let us do this for the marginal probabilities $p_{a},p_{b}$, which by assumption are the same for both distributions. We form new sets of alternatives consisting of those alternatives with distinct probabilities and the representatives of the equivalence classes of the indifference graphs.

Now we construct a matrix $M^{ab}$ whose rows are indexed by the alternatives {x} for observer A and columns by the alternatives {y} for observer B. Assume, furthermore, that these rows and columns are ordered by their probabilities, so that $x_{i}≺x_{j}$ iff $p_{a}(x_{i})<p_{a}(x_{j})$ where p stands for the appropriate marginal probability and ≺ is the order along the row. Similarly, columns follow $y_{i}≺y_{j}$ iff $p_{b}(y_{i})<p_{b}(y_{j})$. We set $M_{xy}^{ab}=p_{a}(x)p_{b}(y)$. Every alternative probability distribution can be obtained as a perturbation of the form $m_{xy}={\tilde{M}}_{xy}^{ab}-M_{xy}^{ab}$, where ${\tilde{M}}_{xy}^{ab}$ is the matrix corresponding to the alternative probability distribution. This is possible because the preference orderings must be identical so that the ordering of rows and columns must be invariant. Since the null sets are isomorphic, they have identical null sets of alternatives. The indifference sets are also identical, and the probabilities can be found by dividing the probability assigned to the representative by the size of its equivalence class. The other probabilities come directly from the matrix.

Boole introduced a set of constraints, ‘conditions of possible experience’, which a probability distribution must satisfy if the distribution is non-contextual [63,64]. There are a number of conditions which must be satisfied here as well if the perturbation is to be valid. These are not the same as Boole’s conditions but possess a similar character. In particular, we have

(i) $0<M_{xy}^{ab}\leq 1$ for all x,y,
(ii) $\sum_{x}\sum_{y}{\tilde{M}}_{xy}^{ab}=1,$
(iii) $\sum_{x}{\tilde{M}}_{xy}^{ab}=\sum_{x}M_{xy}^{ab}=p_{b}(y)$ for all y (consistent connectedness),
(iv) $\sum_{y}{\tilde{M}}_{xy}^{ab}=\sum_{y}M_{xy}^{ab}=p_{a}(x)$ for all x (consistent connectedness),
(v) ${\tilde{M}}_{xy}^{ab}<\min\{p_{a}(x),p_{b}(y)\}$ for all x,y (follows from consistent connectedness and positivity),
(vi) ${\tilde{M}}_{xy}^{ab}<{\tilde{M}}_{x^{\prime }y^{\prime }}^{ab}$ iff $M_{xy}^{ab}<M_{x^{\prime }y^{\prime }}^{ab}$ for all $x,x^{\prime },y,y^{\prime }$ (invariance of order).

The final constraint can equally be written as $({\tilde{M}}_{xy}^{ab}-{\tilde{M}}_{x^{\prime }y^{\prime }}^{ab})(M_{xy}^{ab}-M_{x^{\prime }y^{\prime }}^{ab})>0$ for all $x,x^{\prime },y,y^{\prime }$.

These in turn imply that

(i) $\sum_{x}\sum_{y}m_{xy}=\sum_{x}m_{xy}=\sum_{y}m_{xy}=0$ for all x,y,
(ii) $(m_{xy}-m_{x^{\prime }y^{\prime }})(M_{xy}^{ab}-M_{x^{\prime }y^{\prime }}^{ab})>)$ for all $x,x^{\prime },y,y^{\prime }.$

These constraints are quite strong and do not allow much wiggle room. Note that if one attempts to increase the probability associated with one pair of alternatives, then the values associated with alternatives in the corresponding row and column will, in general, decrease in value to accommodate this. That will decrease the entropy associated with this probability distribution, which is itself of low probability. This suggests the conjecture (though a proof is needed) that the perturbed distribution will not depart significantly from the original product distribution, and so neither will its associated expectation value. If this conjecture is true, then the only ways in which Type II contextuality arises is via either a loss of completeness or of transitivity. Indeed, when one examines intransitivity in preferences in decision making, one follows a chain of paired preferences, assuming naïvely that they all correspond to the same underlying probability distribution, until suddenly one is faced with a contradiction, an intransitivity. In essence, one attempts to introduce a preference relation from one distribution into another, under the mistaken assumption that they are the same distribution. That is the situation given above. The distribution $p_{a,b}$ is consistent in itself, but inconsistent with the presumed joint distribution $p_{a}p_{b}$.

Another way to examine this is to create several bipartite graphs. Given observables $\tilde{A}=\left\{a\right\}$, $\tilde{B}=\left\{b\right\}$, let the vertex set be $M=\tilde{A}\cup \tilde{B}$ and set an edge {a,b} if the probability measure for O(A)[a]⊠Π⊠O(B)[b] is a product measure $p_{a}p_{b}$. We call this graph, $\tilde{M}$, the measure graph for O(A)[a]⊠Π⊠O(B)[b]. We may also define positive and negative correlation graphs $\tilde{P},\tilde{N}$, respectively on M by setting an edge {a,b} in $\tilde{P}$ if E(a,b)>0 and in $\tilde{N}$ if E(a,b)<0. The argument presented previously shows that if a situation is contextual, then it must be the case that in $\tilde{M}$, and in one of $\tilde{P}$ or $\tilde{N}$, there exists a chain which closes, meaning that one has $(a_{1},b_{1}),(b_{1},a_{2}),(a_{2},b_{2}),\ldots ,\left(a_{n},b_{n}\right)$ and $\left(b_{n},a_{1}\right)$, which forms a cycle but which also violates transitivity. If such a chain of observables exists, then there is a good possibility that one will observe true contextuality. This condition is necessary if a chain violates the inequality at a level close to the Tsirelson bound. Each term in the inequality corresponds to an edge in one of the graphs $\tilde{P}$ or $\tilde{N}$ (cases of 0 correlation will not contribute to violations of the inequality, obviously, and can be ignored) and any term with a product measure will contribute an edge to $\tilde{M}$. If the situation is to come close to violating the Tsirelson bound, we know that at some term, the sign of the correlation must switch, which will leave a gap when trying to form the chain in one of $\tilde{P}$ or $\tilde{N}$. Similarly, we know that at some point there must be a term which does not have a product measure, and so this will also leave a gap. Again, we see that relative to the case of a joint product measure, there will be gaps in some chains. Unfortunately, while these conditions appear to be necessary, they are not necessarily sufficient.

## 7. Conclusion

Type II contextuality, usually associated with entanglement, is a hot topic these days thanks to interest in quantum computing. It is frequently attributed to the presence of non-locality, but its discovery in human decision making suggests the need for a more mundane explanation. Reframing the problem within the generative worldview of process and the process algebra suggests that one should look for Type II contextuality in situations in which there exist either a loss of completeness or of transitivity in the preference graph for its dynamics. A loss of completeness would appear to result from the extensionality of the generating processes, which in turn imposes constraints upon measurement processes. Intransitivity would appear to arise from the generative nature of process events, which avoids an absolute link between events and measured values and allows for event values which are variable from event to event and which are conditioned. The interested reader to directed to [2,3,14]. The results presented here are still in an early stage of development but hopefully these ideas will stimulate further research. Future work involves attempting to ascertain those dynamical characteristics or signatures which provide conditions for the appearance of incompleteness and/or intransitivity. An interesting question for future research is the relationship between violations of the triangle inequality, contextuality and transitivity [65], but space limits do not permit its elaboration here.

## Data Availability

This article has no additional data.
